# Detection of PCV2e strains in Southeast China

**DOI:** 10.7717/peerj.4476

**Published:** 2018-03-27

**Authors:** Jiankui Liu, Chunhua Wei, Ailing Dai, Zhifeng Lin, Kewei Fan, Jianlin Fan, Jiayue Liu, Manlin Luo, Xiaoyan Yang

**Affiliations:** 1 College of Life Sciences, Longyan University, Longyan, China; 2 Fujian Provincial Key Laboratory for the Prevention and Control of Animal Infectious Diseases and Biotechnology, Longyan University, Longyan, China; 3 College of Veterinary Medicine, South China Agricultural University, Guangzhou, China

**Keywords:** Phylogenetic analysis, Recombination, PCV2, Genotype

## Abstract

Porcine circovirus 2 (PCV2) has been prevalent in swine herds in China since 2002, causing severe economic loss to the pig industry. The number of live pigs in southeast China is > 20 million. Since information on the genetic variation of PCV2 in the Fujian province is limited, the objective of the present work was to investigate the epidemiological and evolutionary characteristics of PCV2 in southeast China from 2013 to 2017. Of the 685 samples collected from 90 different swine herds from 2013 to 2017, 356 samples from 84 different swine herds were positive for PCV2. PCV2a, PCV2b, PCV2d, and PCV2e co-existed in the Fujian province, with PCV2d being the predominant circulating strain in swineherds and PCV2e being reported for the first time in China. Strikingly, PCV2-FJ-water DNA comes from contaminated river water and not infected animals. Sequence comparison among all isolates indicated that 95 isolates shared approximately 78.7%–100% nucleotide identity and 74.5%–100% amino acid identity for open reading frame 2 (ORF2). Amino acid alignment showed that the Cap protein of PCV2e differed markedly from those of PCV2a, PCV2b, PCV2c, and PCV2d. These results indicated that various PCV2 genotypes exist in China, and that PCV2 is continuously evolving, leading to rapid emergence of new variant stains.

## Introduction

The porcine circovirus (PCV) is a small non-enveloped single-stranded circular DNA virus of swine. Currently, three major PCV2 genotypes have been recognized: PCV type 1 (PCV1), PCV2 type 2 (PCV2), and PCV type 3 (PCV3) (Opriessnig, Meng & Halbur, 2007; Palinski et al., 2016). PCV1 is non-pathogenic to pigs, whereas PCV2 is the primary causative agent of porcine circovirus-associated disease (PCVAD) in growing pigs, which includes systemic, respiratory and enteric manifestations, and PCV3 is associated with porcine dermatitis and nephropathy syndrome (PDNS) (Opriessnig, Meng & Halbur, 2007).

Currently, PCV2 is the primary causative agent of swine viral disease worldwide, which has resulted in considerable economic loss since it was first identified in Canada in 1991 (Harding & Clark, 1997; Krakowka et al., 2000; Bolin et al., 2001). The viral genomes range from 1,766 to 1,768 nucleotides (nt) in length and contain four major identified open reading frames (ORFs) (ORF1–ORF4). ORF2 encodes the capsid protein (Cap), which is the only structural protein of PCV2 and is related to viral antigenicity; hence, the gene encoding the Cap protein is suitable as a phylogenetic and epidemiological marker (Gomes et al., 2007; Choi & Chae, 2008; Jiang et al., 2017). Currently, at least five distinct genotypes have been described in swine based on the ORF2 sequence, which have been classified as PCV2a, PCV2b, PCV2c, PCV2d, and PCV2e (Segalés et al., 2008; Harmon et al., 2015; Xiao, Halbur & Opriessnig, 2015; Davies et al., 2016; Karuppannan & Opriessnig, 2017). PCV2e is a new genetic group which was recently proposed by Davies et al. (2016). Currently, PCV2a, PCV2b, and PCV2d are found globally, whereas PCV2c has only been identified in Denmark and Brazil (Dupont et al., 2008; Franzo et al., 2015). PCV2e is a new genotype that has been circulating in the USA since 2015, the ORF2 sequences of which have 12 or 15 additional nts compared to those of PCV2a–PCV2d (Harmon et al., 2015; Davies et al., 2016).

PCV2 was first reported in China in 1999, and has become the causative agent of one of the country’s most serious swine diseases (Lang et al., 2000; Shuai et al., 2007; Wang et al., 2009; Guo et al., 2012; Jiang et al., 2017). Previous studies showed that various genotypes, including PCV2a, PCV2b, and PCV2d are present in China. Among these genotypes, PCV2b and PCV2d have been predominant in China since 2004 (Lang et al., 2000; Shuai et al., 2007; Wang et al., 2009; Guo et al., 2012; Jiang et al., 2017). Recently, several groups showed that PCV2 isolates in pig farms had recombined owing to the co-existence of different PCV2 genotypes (Hesse, Kerrigan & Rowland, 2008; Cai et al., 2011; Huang et al., 2013; Ramos et al., 2013; Anoopraj et al., 2015). Despite continuous reports of newly emerging strains worldwide, information on the genetic variation of PCV2 in the Fujian province is limited. Therefore, to understand the current molecular epidemiology of PCV2 in Southeast China, the objective of the present work was to investigate the epidemiological and evolutionary characteristics of PCV2 in the Fujian province from 2013 to 2017.

## Materials and Methods

### Animal ethics

Sampling procedures were approved by the Animal Ethics Committee of the South China Agricultural University, with a reference number of SCAU-AEC-2014-10.

### Clinical samples

**Figure 1:**
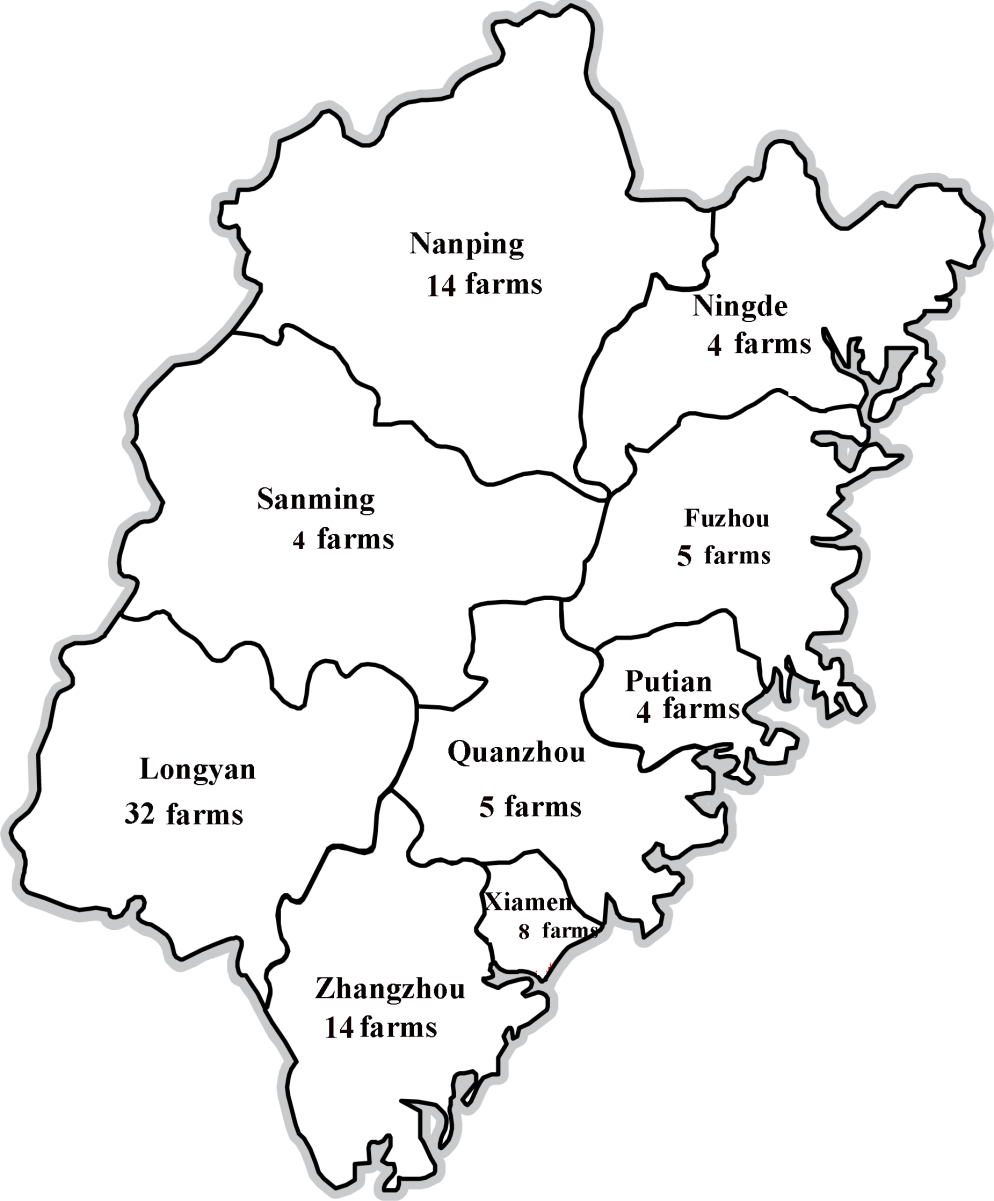
Locations of PCV2-positive swine farms where samples were collected in Fujian Province.

Six hundred and eighty-five clinical samples (blood, lungs, kidneys, livers, and lymph nodes) were collected from unthrifty pigs (4–10 weeks of age) in different farms of Fujian province, China, between 2013 and 2017. In particular, samples were collected from 32 farms in Longyan (196 samples), 14 farms in Zhangzhou (105 samples), eight farms in Xiamen (75 samples), five farms in Fuzhou (54 samples), four farms in Ningde (40 samples), 14 farms in Nanping (92 samples), four farms in Sanming (40 samples), five farms in Quanzhou (45 samples), and four farms in Putian (38 samples) from 2013 to 2017 (Fig. 1). These pigs were suspected to have clinical signs of Postweaning Multisystemic Wasting Syndrome (PMWS) and/or PDNS. In addition, 200 water samples were collected from the river or pool covering a geographic area of about 5 km^2^ near every pig farm.

### Polymerase chain reaction (PCR) amplification and nucleotide sequencing

Total viral DNA was extracted from tissue samples using DNA extraction kit ver.3.0 (TaKaRa, Dalian, China) following the manufacturer’s protocol. ORF2 was amplified from the extracted DNA using specific primers according to previous studies (Fort et al., 2007). PCR products were purified using the gel extraction kit (Tiangen Biotech Co. Ltd., Beijing, China) and cloned into the pGEM-T Easy vector (Promega). Recombinant clones were sequenced by Sangon Biotech (Shanghai) Co. Ltd. Each fragment was independently sequenced at least thrice.

### Phylogenetic analysis

Ninety-five ORF2 genes of PCV2 isolates were identified in the Fujian province during 2013–2017. Representative PCV2 sequences, including PCV2a, PCV2b, PCV2c, PCV2d, and PCV2e isolates in the GenBank, were used for sequence alignments and phylogenetic analyses.

Multiplex sequencing alignments were performed using CLUSTAL X (version 1.83). The phylogenetic tree was constructed using the neighbor joining (NJ) method with MEGA 6.0, the maximum composite likelihood model, and a bootstrap confidence value of 1,000 replicates.

### Recombination analysis

To detect putative recombination events in PCV2, recombinant strains were investigated on the complete genome database using a recombination detection program (RDP v.4.24) (Martin et al., 2010) as described by Ramos et al. (2013). Briefly, seven methods (RDP, GeneConv, BootScan, MaxChi, Chimera, SiScan, and 3Seq) were used for preliminary scan and only events detected by more than two methods with a highest acceptable *p*-value of 0.01 were considered. The recombination event was further confirmed using SimPlot program (v.3.5.1) and boot scanning analysis was performed with a 200-bp window sliding along a step size of 20 bp (Lole et al., 1999).

## Results

### PCV2 detection

Of the 685 samples collected from 90 different swine herds located in Fujian province from 2013 to 2017, 356 samples from 84 different swine herds were positive for PCV2. Ninety-five positive samples were selected for further analysis of PCV2 characteristics (Table 1).

### Phylogenetic analysis of ORF2

**Figure 2:**
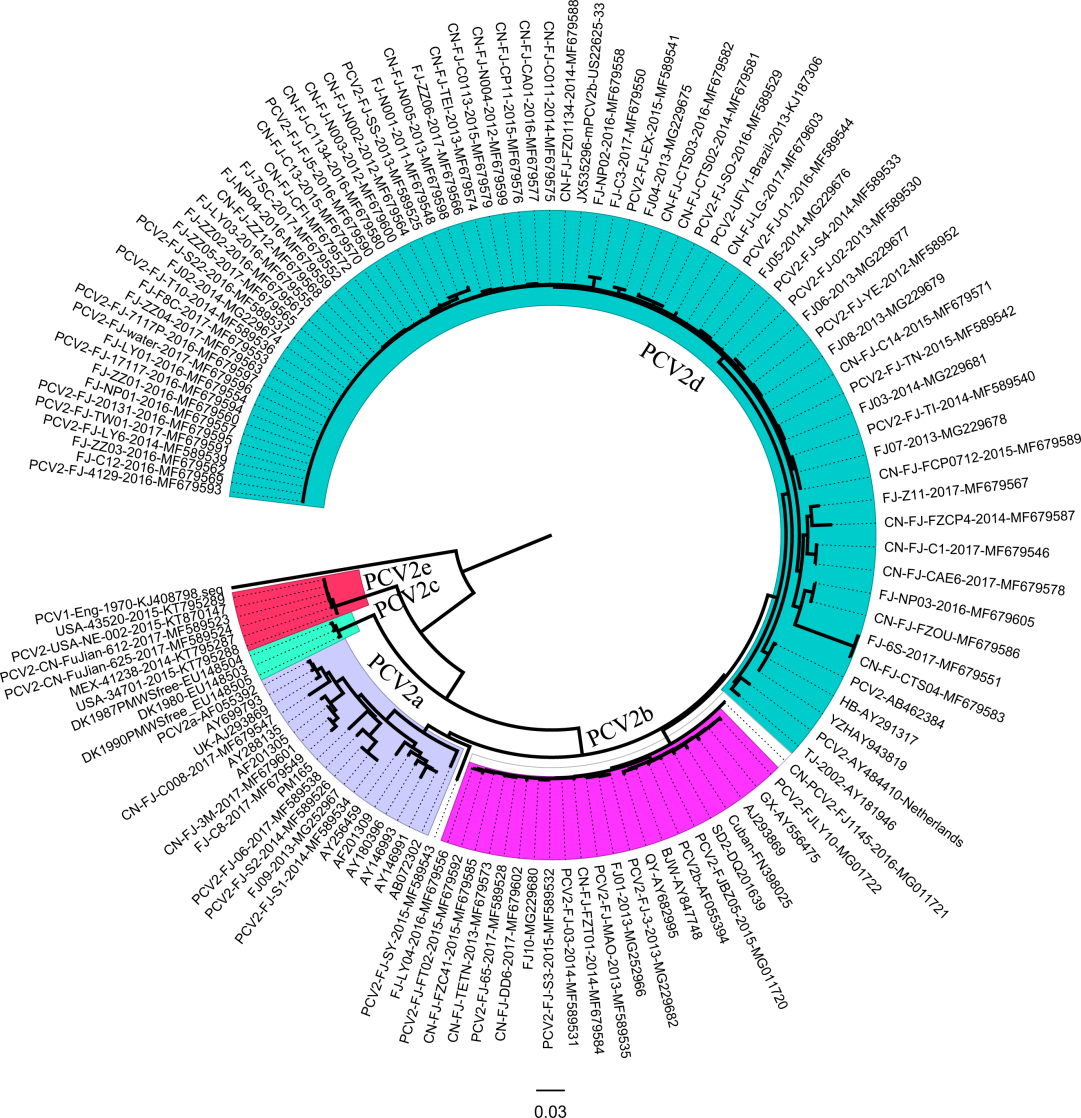
Neighbor-joining (NJ) phylogenetic analysis of Fujian PCV2 ORF2 genes. Reliability of the tree was assessed by bootstrap analysis of 1,000 replications. PCV2b (GenBank accession no. AF055394).

Phylogenetic analysis based on ORF2 showed that Fujian PCV2 strains were classified into five subtypes (PCV2a, PCV2b, PCV2d, and PCV2e) (Fig. 2). Among Fujian PCV2 strains, 7.37% (7/95) of the sequences corresponded to PCV2a, 15.8% (15/95) to PCV2b, 72.6% (69/95) to PCV2d, and 2.1% (2/95) to PCV2e. PCV2-CN-FuJian-612-2017 and PCV2-CN-FuJian-625-2017 were classified as PCV2e, which has been reported for the first time in China. Interestingly, PCV2-FJ-water DNA comes from contaminated water instead of from infected animals.

**Table 1:** List of Fujian PCV2 samples in this study.

Name	Geographic origin	Source	Time	Genetype	Accession No.
CN-FJ-C1-2017	Putian	lymph node	2017	PCV2d	MF679546
CN-FJ-C008	Putian	serum	2017	PCV2a	MF679547
CN-FJ-N001-2011	Longayan	lymph node	2011	PCV2d	MF679548
CN-FJ-C8-2017	Longayan	Spleen/Lung	2017	PCV2a	MF679549
CN-FJ-C3-2017	Longayan	lymph node	2017	PCV2d	MF679550
CN-FJ-6S-2017	Zhangzhou	lymph node	2017	PCV2d	MF679551
CN-FJ-7SC-2017	Putian	serum	2017	PCV2d	MF679552
CN-FJ-F8C-2017	Xiamen	lymph node	2017	PCV2d	MF679553
CN-FJ-LY01-2016	Quanzhou	Lung	2016	PCV2d	MF679554
CN-FJ-LY03-2016	Ningde	lymph node	2016	PCV2d	MF679555
PCV2-FJ-3	Ningde	serum	2013	PCV2b	MG229682
FJ01	Longyan	lymph node	2013	PCV2a	MG252966
FJ02	Zhangzhou	Spleen/Lung	2014	PCV2d	MG229674
FJ03	Xiamen	lymph node	2014	PCV2d	MG229681
FJ04	Nanping	lymph node	2013	PCV2d	MG229675
FJ05	Fuzhou	lymph node	2014	PCV2d	MG229676
FJ06	Longyan	serum	2013	PCV2d	MG229677
FJ07	Quanzhou	lymph node	2013	PCV2d	MG229678
FJ08	Zhangzhou	Lung	2013	PCV2d	MG229679
FJ09	Longyan	lymph node	2013	PCV2b	MG252967
FJ10	Nanping	lymph node	2014	PCV2b	MG229680
CN-FJ-LY04-2016	Quanzhou	serum	2016	PCV2b	MF679556
CN-FJ-NP01-2016	Nanping	lymph node	2016	PCV2d	MF679557
CN-FJ-NP02-2016	Nanping	Spleen/Lung	2016	PCV2d	MF679558
CN-FJ-NP03-2016	Nanping	lymph node	2016	PCV2d	MF679605
FJ-NP04-2017	Nanping	serum	2016	PCV2d	MF679559
CN-FJ-ZZ01-2016	Zhangzhou	lymph node	2016	PCV2d	MF679560
CN-FJ-ZZ02-2016	Zhangzhou	Spleen/Lung	2016	PCV2d	MF679561
CN-FJ-ZZ03-2016	Zhangzhou	lymph node	2016	PCV2d	MF679562
CN-FJ-ZZ04-2017	Zhangzhou	Lung	2017	PCV2d	MF679563
CN-FJ-N002-2012	Quanzhou	lymph node	2012	PCV2d	MF679564
CN-FJ-ZZ05-2017	Zhangzhou	spleen	2017	PCV2d	MF679565
CN-FJ-ZZ06-2017	Zhangzhou	lymph node	2017	PCV2d	MF679566
CN-FJ-C011	Xiamen	serum	2015	PCV2d	MF679575
CN-FJ-Z11	Xiamen	lymph node	2017	PCV2d	MF679567
CN-FJ-ZZ12	Xiamen	serum	2015	PCV2d	MF679568
CN-FJ-C12	Xiamen	lymph node	2016	PCV2d	MF679569
CN-FJ-C13	Quanzhou	Lung	2015	PCV2d	MF679570
CN-FJ-C14	Longyan	Spleen/Lung	2015	PCV2d	MF679571
CN-FJ-CFI	Longyan	lymph node	2013	PCV2d	MF679572
CN-FJ-TETN	Longyan	serum	2013	PCV2b	MF679573
CN-FJ-TEI	Quanzhou	serum	2013	PCV2d	MF679574
CN-FJ-CP11	Quanzhou	lymph node	2015	PCV2d	MF679576
CN-FJ-CA01	Quanzhou	lymph node	2016	PCV2d	MF679577
CN-FJ-CAE6	Quanzhou	spleen	2017	PCV2d	MF679578
CN-FJ-C0113	Zhangzhou	lymph node	2015	PCV2d	MF679579
CN-FJ-C1134	Xiamen	serum	2016	PCV2d	MF679580
CN-FJ-CTS02	Longyan	lymph node	2014	PCV2d	MF679581
CN-FJ-CTS03	Longyan	lymph node	2016	PCV2d	MF679582
CN-FJ-CTS04	Longyan	Spleen/Lung	2015	PCV2d	MF679583
CN-FJ-FZT01	Xiamen	lymph node	2014	PCV2b	MF679584
CN-FJ-FZC41	Quanzhou	serum	2015	PCV2b	MF679585
CN-FJ-FZOU	Ningde	lymph node	2013	PCV2d	MF679586
CN-FJ-FZCP4	Ningde	lymph node	2014	PCV2d	MF679587
CN-FJ-FZ01134	Ningde	Spleen/Lung	2014	PCV2d	MF679588
CN-FJ-FCP0712	Ningde	lymph node	2015	PCV2d	MF679589
PCV2-FJ-FJ5	Quanzhou	spleen	2016	PCV2d	MF679590
PCV2-FJ-TW01	Quanzhou	lymph node	2017	PCV2d	MF679591
PCV2-FJ-FT02	Putian	lymph node	2015	PCV2b	MF679592
PCV2-FJ-4129	Putian	Spleen/Lung	2016	PCV2d	MF679593
PCV2-FJ-17117	Putian	lymph node	2016	PCV2d	MF679594
PCV2-FJ-20131	Putian	lymph node	2016	PCV2d	MF679595
PCV2-FJ-water	Longyan	Water	2017	PCV2d	MF679596
PCV2-FJ-7117P	Putian	lymph node	2016	PCV2d	MF679597
CN-FJ-N005-2013	Zhangzhou	serum	2013	PCV2d	MF679598
CN-FJ-N004-2012	Fuzhou	lymph node	2012	PCV2d	MF679599
CN-FJ-N003-2012	Nanping	Lung	2012	PCV2d	MF679600
CN-FJ-3M-2017	Nanping	lymph node	2017	PCV2a	MF679601
CN-FJ-DD6-2017	Fuzhou	lymph node	2017	PCV2b	MF679602
CN-FJ-LG-2017	Longyan	Lung	2017	PCV2d	MF679603
PCV2-CN/FuJian-612-2017	Longyan	lymph node/spleen	2017	PCV2e	MF589523
PCV2-CN/FuJian-625-2017	Longyan	lymph node/spleen	2017	PCV2e	MF589524
PCV2-FJ-SS	Fuzhou	lymph node	2013	PCV2d	MF589525
PCV2-FJ-S2	Nanping	Spleen/Lung	2014	PCV2a	MF589526
PCV2-FJ-YE	Longyan	lymph node	2012	PCV2d	MF589527
PCV2-FJ-65	Longyan	Lung	2017	PCV2b	MF589528
PCV2-FJ-SO	Longyan	lymph node	2016	PCV2d	MF589529
PCV2-FJ-02	Longyan	Spleen/Lung	2013	PCV2d	MF589530
PCV2-FJ-03	Longyan	lymph node	2014	PCV2b	MF589531
PCV2-FJ-S3	Longyan	serum	2015	PCV2b	MF589532
PCV2-FJ-S4	Longyan	lymph node	2014	PCV2d	MF589533
PCV2-FJ-S1	Fuzhou	lymph node	2014	PCV2a	MF589534
PCV2-FJ-MAO	Fuzhou	Lung	2013	PCV2b	MF589535
PCV2-FJ-T10	Fuzhou	lymph node	2014	PCV2d	MF589536
PCV2-FJ-S22	Fuzhou	Spleen/Lung	2016	PCV2d	MF589537
PCV2-FJ-06	Fuzhou	Lung	2017	PCV2a	MF589538
PCV2-FJ-LY6	Zhangzhou	lymph node	2014	PCV2d	MF589539
PCV2-FJ-TI	Zhangzhou	Spleen	2014	PCV2d	MF589540
PCV2-FJ-EX	Zhangzhou	lymph node	2015	PCV2d	MF589541
PCV2-FJ-TN	Zhangzhou	lymph node	2015	PCV2d	MF589542
PCV2-FJ-SY	Longyan	lymph node	2015	mPCV2d	MF589543
PCV2-FJ-01	Quanzhou	serum	2016	PCV2d	MF589544
PCV2-FJBZ05	Longyan	lymph node	2015	PCV2b	MG011720
CN-PCV2-FJ1145	Longyan	Spleen/Lung	2016	mPCV2b	MG011721
PCV2-FJLY10	Longyan	lymph node	2016	PCV2b	MG011722

### Amino acid analysis of ORF2

The lengths of the Fujian PCV2 ORF2 sequences were 702 nt (PCV2a, PCV2b, or PCV2d), 705 nt (PCV2c and PCV2d), and 717 nt (PCV2e), which encoded Cap proteins of 233, 234, and 238 amino acids, respectively. These 95 isolates shared approximately 78.7%–100% nucleotide identity and 74.5%–100% amino acid identity for ORF2. Amino acid alignments showed that the Cap protein of PCV2e and two strains (CN-FJ-CTS04 and FJ-6S-2017) differed markedly from those of PCV2a, PCV2b, PCV2c, and PCV2d. Unlike the sequence of other known PCV2 ORF2, the PCV2e ORF2 possesses 12 or 15 additional nts, which generate a terminal seven amino acid sequence (PPPLSYM) that is different from the PK and NPK termini of the 233 and 234 amino acid capsids, respectively (Davies et al., 2016). The PCV2 strains in genotype PCV2d harbored four unique amino acid mutations (Y^8^ → F/T^8^, A^68^ → N^68^, F^53^ → I^53^, and V^215^ → I^215^), whereas PCV2 in genotype PCV2e harbored ten unique amino acid mutations (K^58^ → V^8^, D^115^ → E^115^, A^133^ → T^133^, A^135^ → N^135^, T^137^ →  S^137^, T^170^ → I^170^, V^196^ → T^196^, N^204^ → H^204^, Y^207^ →  T^207^ and L^226^ → M^226^).

### Recombination analysis

**Figure 3:**
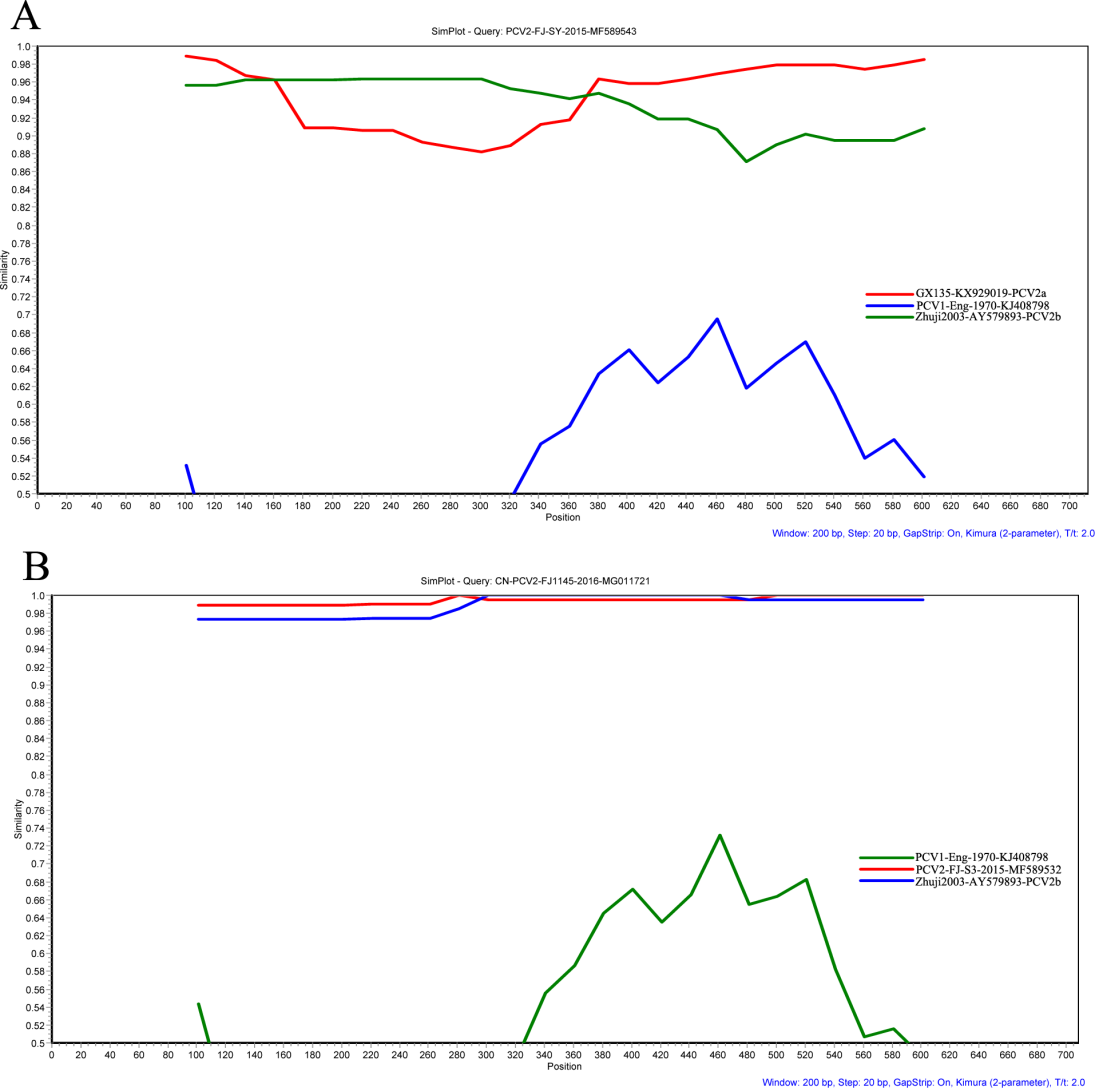
Recombination analysis of the PCV2 ORF2 gene of PCV2-FJ-SY (A) and CN-PCV2-FJ1145 (B) using a sliding window of 200 nt moving in 20 nt steps. Recombination events were analyzed by Simplot software (v. 3.5.1). A PCV1 isolate (KJ408798) was used as outgroup.

Seven methods (RDP, GeneConv, BootScan, MaxChi, Chimera, SiScan, and 3Seq) were implemented in RDP 4.24 to detect potential recombinants. The results showed that inter-genotypic (PCV2-FJ-SY) and intra-genotypic (CN-PCV2-FJ1145) recombination events of PCV2 have been detected (Fig. 3). Additionally, the results of SIMPLOT software (v3.5.1) confirmed the putative recombinant events obtained with the RDP 4.24 software.

## Discussion

PCV2 has been prevalent in Chinese swine herds since it was first identified in China in 1999. Previous studies demonstrated that various PCV2 genotypes exist in China, including PCV2a, PCV2b, PCV2c, PCV2d, and a minor recombinant group. Surprisingly, different PCV2 genotypes or strains have co-existed in Chinese swine herds. Currently, the amount of live pigs in the Fujian province (Southeast China) is >20 million heads, although the epidemic status and genetic diversity of PCV2 is still unknown. Thus, we sought to investigate the genetic diversity and epidemiology of PCV2 in southeast China in 2013–2017.

On the basis of ORF2 nucleotide sequences, the genotypes of Fujian PCV2 have been classified as PCV2a, PCV2b, PCV2d, and PCV2e (Opriessnig, Meng & Halbur, 2007; Guo et al., 2010; Guo et al., 2012; Harmon et al., 2015; Davies et al., 2016), of which, PCV2d was predominant in Fujian from 2014 to 2017. The present data indicate that the prevalence of PCV2d increased from 57.1% in 2013 to 87.5% in 2016, suggesting that PCV2d has replaced PCV2b and has now become the predominant PCV2 genotype in Fujian since 2013. Additionally, CN-FJ-CTS04 and FJ-6S-2017 strains emerged in cases of in cases of apparent vaccine failure. Overuse of vaccine has probably contributed to the variation of PCV2. PCV2e, a new PCV2 genotype, was identified in the USA in 2015, the ORF2 sequence of which contains 12 or 15 additional nt compared to other known PCV2 ORF2 sequences and shows approximately 85% identity with other known PCV2 ORF2 sequences (Harmon et al., 2015; Davies et al., 2016). In the present study, only two PCV2e sequences were detected in Fujian, suggesting that PCV2e replication is low in the swine herds. The group members encoded a capsid protein of 238 amino acids, which is markedly different from all known capsid proteins of 233 or 234 amino acids (Xiao et al., 2016). The pathogenicity of PCV2e warrants further investigation.

Recently, several groups detected PCV2 DNA in water samples, human stool, beef, calf bone marrow, farm air, house flies, and mosquito (Verreault et al., 2010; Blunt et al., 2011; Garcia et al., 2012; Yang et al., 2012). In the present study, PCV2 DNA was also detected in river water, which is indicative of a potential human health hazard.

PCV2 nucleotide substitution rate has been calculated to vary between 1.2 × 10^−3^ and 6.6 × 10^−3^ substitutions/site/year, which is close to that of RNA viruses (Firth et al., 2009; Pérez et al., 2011). Recombination is critical for viral evolution, and recombination events between PCV2 strains could generate PCV2 of varying pathogenicity (Olvera, Cortey & Segales, 2007; Hesse, Kerrigan & Rowland, 2008; Wang et al., 2009). Both inter-genotypic and intra-genotypic recombination events of PCV2 have been recorded; inter-genotypic recombination occurs within ORF1 and ORF2, whereas intra-genotypic recombination mainly occurs within ORF2. Recently, a PCV2c genotype strain (GenBank accession no. KC823058) was reported in China, and three strains were generated from PCV2b and PCV2c recombination (Liu et al., 2016). In the present study, we detected that one natural strain (PCV2-FJ-SY) was generated by recombination between different lineages of PCV2, while CN-PCV2-FJ1145 was generated by intra-genotypic recombination. These results indicated that PCV2 is continuously evolving through genome recombination, which leads to rapid emergence of new variant stains.

In summary, PCV2a, PCV2b, PCV2d, and PCV2e co-exist in Fujian, and PCV2d-2 is the predominant strain that has been circulating in Fujian since 2013. Our results enhance the understanding of the PCV2 epidemic in Fujian, and may assist veterinary workers in establishing suitable prevention and control policies for the novel strains emerging from viral evolution.

## Conclusions

The findings of this study demonstrated that PCV2 is ubiquitous in China, with PCV2d being the predominant strain circulating from 2013 to 2017. PCV2 is continuously evolving through genome recombination or new viral introductions from distant geographic regions, which leads to rapid emergence of new variant stains.

##  Supplemental Information

10.7717/peerj.4476/supp-1 Supplemental Information 1 New sequences

## Additional Information and Declarations

### Field Study Permissions

The following information was supplied relating to field study approvals (i.e., approving body and any reference numbers): Sampling procedures were approved by the Animal Ethics committee of the South China Agricultural University, reference number SCAU-AEC-2014-10.

### DNA Deposition

The following information was supplied regarding the deposition of DNA sequences: GenBank: MF589523, MF589524.

### Data Availability

The following information was supplied regarding data availability: Sequence data can be accessed through the GenBank accession numbers in Table 1 and in the Supplemental Information.
