# Supplementary material for: Detection of PCV2e strains in Southeast China
Source: PeerJ. 2018 Mar 27;6:e4476. doi: 10.7717/peerj.4476 (PMC5877446; doi:10.7717/peerj.4476)
Supplement: Supplemental Information 1 [file peerj-06-4476-s001.docx]

**Porcine circovirus 2 strain PCV2-CN/FuJian-612-2017, complete genome**

accagcgcacttcggcagcggcagcacctcggcagcacctcagcagcaaaatgcccagcaagaagaatggaagaagcgga

ccccaaccacacaaaaggtgggtgttcacgctgaataatccttccgaagacgagcgcaagaaaatacgggagcttccaat

ctccctttttgattattttattgttggcgaggagggtaatgaggaaggacgaacaccccacctccaggggttcgctaatt

ttgtgaagaagcaaacattcaataaagtgaaatggtattttggtgcccgctgccacatcgagaaagcgaaaggaaccgat

catcagaataaagaatattgcagtaaagaaggcaacttactgattgaatgtggagctcctagatctcaaggacaacggag

tgacctgtctactgctgtgagtaccttgttggagagcgggagtctggtgaccgttgcagagcagcaccctgtaacgtttg

tcagaaatttccgcgggctggctgaacttttgaaagtgagcgggaaaatgcagaagcgtgattggaagacgaatgtacac

gtcattgtggggccacctgggtgtggcaaaagcaaatgggctgctaattttgcagacccggaaaccacatattggaaacc

acctagaaacaagtggtgggatggttaccatggtgaagaagtggttgttattgatgacttttatggctggctgccgtggg

atgatctactgagactctgtgatcgatatcctttgactgttgagactaaaggtggaactgtaccttttttggcccgcagt

attctgattaccagcaatcagaccccgttggaatggtactcctcaactgctgtcccagctgtagaagctctctatcggag

gattacttccttggtattttggaagaatgctacagaacaatccacggaggaagggggccagttcgtcaccctttcccccc

catgccctgaatttccttatgaaataaattactgagtcttgtatcacttcgtaatggtttttattttttacatataagaa

agaggggaaggtagaggggggtctttcatattaaattctctaaattgtacgtagagagttacacgtacattgtaggcctg

ggcagtggtactgtgttgaaaagcagtgccaaggcctgtatggtccacattaccagaggtttgtagtctcagccagagtt

gatttcttttgttattgggttggaagtaatcgattatcctatcaaggacaggtttgggggtgaaatatcgggagtggtat

gagaagggctgggttatggtgtggcgggaggagtagtttacataggggtcgtaggacaggttattggttttgtttacgaa

gttatcatctaagattacagcactggagcctactccccgttcaccttgagtgatgggggatcttgcaaagaattctactt

taacctttcttattctgtagtattcgaagggtacagagagggggtttgttccccctcctggtgggaggaaatctccaata

ttaaatctcatcatgtccaccgcccatgatggcggtgtgactgtggaggttacaacagtatatacaaaggagcgggagag

gcgggcgttgaagattccatttttccttctccagcggtaacggtggcgggggtggacgagccaggggcggcggcggagga

tctggccaagatggctgcgggggcggtgtcttctccttcggtaacgcctccttggatacgtcatagctgaaaacgaaaga

agtgcgctgtaagtatt

**Porcine circovirus 2 strain PCV2-CN/FuJian-625-2017, complete genome**

accagcgcacttcggcagcggcagcacctcggcagcacctcagcagcaaaatgcccagcaagaagaatggaagaagcgga

ccccaaccacacaaaaggtgggtgttcacgctgaataatccttccgaagacgagcgcaagaaaatacgggagcttccaat

ctccctttttgattattttattgttggcgaggagggtaatgaggaaggacgaacaccccacctccaggggttcgctaatt

ttgtgaagaagcaaacattcaataaagtgaaatggtattttggtgcccgctgccacatcgagaaagcgaaaggaaccgat

catcagaataaagaatattgcagtaaagaaggcaacttactgattgaatgtggagctcctagatctcaaggacaacggag

tgacctgtctactgctgtgagtaccttgttggagagcgggagtctggtgaccgttgcagagcagcaccctgtaacgtttg

tcagaaatttccgcgggctggctgaacttttgaaagtgagcgggaaaatgcagaagcgtgattggaagacgaatgtacac

gtcattgtggggccacctgggtgtggcaaaagcaaatgggctgctaattttgcagacccggaaaccacatattggaaacc

acctagaaacaagtggtgggatggttaccatggtgaagaagtggttgttattgatgacttttatggctggctgccgtggg

atgatctactgagactctgtgatcgatatcctttgactgttgagactaaaggtggaactgtaccttttttggcccgcagt

attctgattaccagcaatcagaccccgttggaatggtactcctcaactgctgtcccagctgtagaagctctctatcggag

gattacttccttggtattttggaagaatgctacagaacaatccacggaggaagggggccagttcgtcaccctttcccccc

catgccctgaatttccttatgaaataaattactgagtcttgtatcacttcgtaatggtttttattttttacatataagaa

agaggggaaggaagaggggggtctttcatattaaattctctaaattgtacgtagagagttacacgtacattgtaggcctg

ggcagtggtactgtgttgaaaagcagtgccaaggcctgtatggtccacattaccagaggtttgtagtctcagccagagtt

gatttcttttgttattgggttggaagtaatcgattatcctatcaaggacaggtttgggggtgaaatatcgggagtggtat

gagaagggctgggttatggtgtggcgggaggagtagtttacataggggtcgtaggacaggttattggttttgtttacgaa

gttatcatctaagattacagcactggagcctactccccgttcaccttgagtgatgggggatcttgcaaagaattctactt

taacctttcttattctgtagtattcgaagggtacagagagggggtttgttccccctcctggtgggaggaaatctccaata

ttaaatctcatcatgtccaccgcccatgatggcggtgtgactgtggaggttacaacagtatatacaaaggagcgggagag

gcgggcgttgaagattccatttttccttctccagcggtaacggtggcgggggtggacgagccaggggcggcggcggagga

tctggccaagatggctgcgggggcggtgtcttctccttcggtaacgcctccttggatacgtcatagctgaaaacgaaaga

agtgcgctgtaagtatt
